# Climate change and mosquito-borne diseases in China: a review

**DOI:** 10.1186/1744-8603-9-10

**Published:** 2013-03-09

**Authors:** Li Bai, Lindsay Carol Morton, Qiyong Liu

**Affiliations:** 1 State Key Laboratory for Infectious Diseases Prevention and Control, National Institute for Communicable Disease Control and Prevention, Chinese Center for Disease Control and Prevention, Beijing 102206, People’s Republic of China; 2 University of South Florida College of Public Health, 4202 E. Fowler Avenue, Tampa, FL, 33620, USA; 3 Shandong University Climate Change and Health Center, Jinan, Shandong 250012, People’s Republic China

**Keywords:** Climate change, Malaria, Dengue fever, Japanese encephalitis, Adaptation, China

## Abstract

China has experienced noticeable changes in climate over the past 100 years and the potential impact climate change has on transmission of mosquito-borne infectious diseases poses a risk to Chinese populations. The aims of this paper are to summarize what is known about the impact of climate change on the incidence and prevalence of malaria, dengue fever and Japanese encephalitis in China and to provide important information and direction for adaptation policy making. Fifty-five papers met the inclusion criteria for this study. Examination of these studies indicates that variability in temperature, precipitation, wind, and extreme weather events is linked to transmission of mosquito-borne diseases in some regions of China. However, study findings are inconsistent across geographical locations and this requires strengthening current evidence for timely development of adaptive options. After synthesis of available information we make several key adaptation recommendations including: improving current surveillance and monitoring systems; concentrating adaptation strategies and policies on vulnerable communities; strengthening adaptive capacity of public health systems; developing multidisciplinary approaches sustained by an new mechanism of inter-sectional coordination; and increasing awareness and mobilization of the general public.

## Introduction

The Intergovernmental Panel on Climate Change (IPCC) has reported the existence of abundant evidence of climate change on a global scale [1]. According to the IPCC’s fourth assessment in 2007, global average surface temperature will increase by 1.1-6.4°C by 2100, 2-9 times more than globally averaged warming during last century [1]. Furthermore, the frequency and extent of extreme weather events; such as heat waves, bushfires, floods, and cyclones, can be highly impacted by the changing climate. Anthropogenic climate change has also been identified as an important risk factor for population health [2], including transmission of infectious diseases, and most importantly suspected impacts distribution and occurrence of vector borne diseases [1]. Despite the ongoing debate over the influence climate factors have on mosquito-borne disease occurrence, it is widely assumed that distribution and occurrence of these diseases, such as malaria, are determined by climate and that global warming trends will lead to higher incidence and wider geographic range [3-10]. In contrast, some studies hold that the current evidence is insufficient to clearly attribute local resurgences or such geographic spread to regional changes in climate [11-14]. More research is needed to better understand the relationship between climate change and transmission of mosquito-borne diseases, and to further promote adaptive policies formulation to reduce unexpected climate-related risk at a global, regional or local level.

Mosquito-borne diseases in China remain a serious public health problem. For example, 46,988 malaria cases and 18 deaths were reported in 1,097 counties in 2007 [15]. In 2002, the most serious outbreak of dengue fever occurred in Taiwan with 5,285 diagnosed cases [16]. In 2006, an outbreak of Japanese encephalitis occurred in Shanxi Province causing 19 deaths [17]. As the largest developing country, China has experienced considerable changes in climate over during the last decade with more rapid changes in the past 50 years [18]. The annual average temperature has risen by 0.5-0.8°C, which is slightly higher than the global average level. These variation and fluctuation in weather patterns and extreme climatological phenomena (e.g. droughts, storms, floods etc.) may have a detrimental effect on frequency and distribution of mosquito-borne diseases.

In recent years, the impact of climate change on the transmission of mosquito-borne diseases has been studied in China. However, the quantitative relationship between meteorological variables and the spatial and temporal distributions of these infectious diseases is still not clear. Study findings are inconsistent, which may be due to methodological limitations, unavailability of relevant data and many uncertainties about the range and magnitude of influences of climate change. Moreover, there remains no adaptive mechanism to reduce adverse consequences of mosquito-borne diseases under the changing climate in China. It is urgent to improve our understanding of current evidence, knowledge gaps and development of adaptation options. Our aims were to summarize previous research exploring climate change-related impacts on mosquito-borne diseases in China by reviewing the published studies examining the relationship between climate variability and the transmission of malaria, dengue fever and Japanese encephalitis, and to give some suggestions for the development of adaptation strategies and measures to lessen the adverse impacts on mosquito-borne diseases in China.

## Methods

### Search strategy

**Figure 1:**
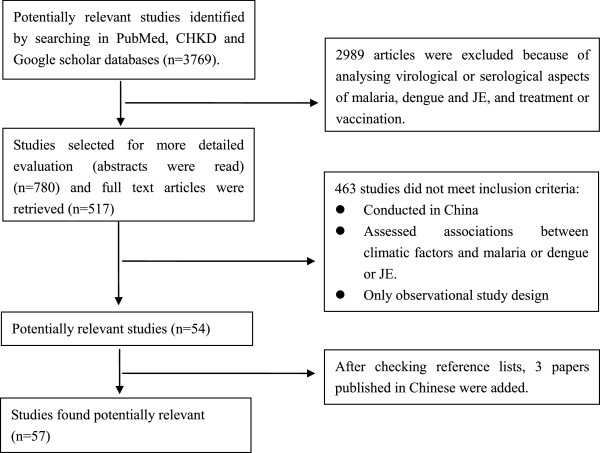
Flow chart of literature search strategy.

The PubMed electronic database and China Hospital Knowledge Database (CHKD) were used in December 2011 to retrieve original studies published in English and Chinese, respectively. Searches of the “Google” search engine and “Google Scholar” were also conducted. Combinations of the key terms “malaria”, “dengue”, “dengue fever”, “dengue hemorrhagic fever”, “Japanese encephalitis”, “climate”, “weather”, “climate change”, “climate variability”, “climatic factors”, “temperature”, “rainfall”, and “humidity” were used to maximize search yield. Titles, abstracts and keywords were first screened for relevance and full texts were obtained to evaluate for inclusion criteria. Reference lists of each included article were then evaluated if missed in the in the initial electronic database search. Figure 1 illustrates the systematic search and inclusion/exclusion process.

### Inclusion criteria

Studies were included on the basis of the following criteria:

1. Articles must evaluate the effects of climatic factors on the distribution and transmission of malaria, dengue fever or Japanese encephalitis. Meteorological variables (e.g. temperature, rainfall etc.) or ecological proxy indicators (e.g. Normalized Difference Vegetation Index, South Oscillation Index) had to be included. Disease variables (e.g. incidence, cases) or entomologic variables (e.g. Breteau Index, House Index etc.) had to be included.

2. The papers had to use an epidemiological study design (e.g. time series analysis, spatial and/or temporal analysis and descriptive study) to identify the association between climatic variables and incidence of mosquito-borne diseases and/or vector factors (e.g. mosquito density, distribution, infectious life span).

3. Only studies published before December 2012 and conducted in China (including Chinese Autonomous Regions, Hong Kong, Macau and Taiwan) were examined.

## Results and discussion

### Literature search

To avoid language bias, studies published in English and Chinese were considered for inclusion (Table 1). The initial search generated 3769 articles from PubMed, CHKD and Google Scholar databases. Review of the titles, abstracts and keywords excluded 2989 articles, leaving 780 studies identified as potential epidemiological papers. Then, 517 full-text articles were identified based on the abstracts and evaluated for inclusion. Of these, fifty-four articles met the inclusion criteria and 3 papers were included after cross-referencing. The methodologies and major findings of the final 57 studies are summarized in Tables 2, 3 and 4.

**Table 1:** Numbers of selected studies published in English and Chinese

	**Studies in English *Published between 1998-2011***	**Studies in Chinese *Published between 1995-2011***	**Total**
Malaria	13	15	**28**
Dengue fever	9	6	**15**
Japanese encephalitis	4	10	**14**
**Total**	**26**	**31**	**57**

**Table 2:** Characteristics of studies on the association between climatic variables and malaria transmission

**Study & Language**	**Study area & period**	**Data Collection**		**Statistical Methods**	**Main findings**	**Comments**
		**Risk factors**	**Disease/vector**			
Huang et al. (2011) English [19]	Anhui, Henan, Hubei Provinces 1990-2009	Normalized annual temperature, relative humidity and rainfall	Cases counts	-Bayesian Poisson models	-Rainfall played a more important role in malaria transmission than other meteorological factors.	-Spatial-temporal models were developed
				- GIS		-Socioeconomic factors were not taken into account.
Huang et al. (2011) English [20]	Motuo County, Tibet 1986-2009	Monthly average temperature, maximum temperature, minimum temperature, relative humidity and total amount of rainfall	Monthly incidence of malaria	-Spearman correlation analysis	-Relative humidity was more sensitive to monthly malaria incidence.	-Several statistical methods were applied
				-Cross-correlation analysis	-The relationship between malaria incidence and rainfall was not directly and linearly.	-Only one county was considered
				-SARIMA model		
				-Inter-annual analysis		
Zhou et al. (2010) English [21]	Huaiyuan County of Anhui and Tongbai County of Henan Province 1990-2006	Monthly and annual average temperature, maximum temperature, minimum temperature, relative humidity and rainfall	Monthly and annual incidence of malaria Vectorial capacity	-Spearman correlation	-Temperature and rainfall were major determinants for malaria transmission. However, no relationship between malaria incidence and relative humidity was observed.	-Entomological investigate was conducted to determine the vectorial effect of malaria re-emergency.
				-Stepwise regression analysis		
				-Curve fitting		
				-Trend analysis		-Only two counties were examined
				- Entomological investigation		
Zhang et al. (2010) English [22]	Jinan city, Shangdong Province 1959-1979	Monthly average maximum temperature, minimum temperature, relative humidity and rainfall	Cases counts	-Spearman correlation	-Temperature was greatest relative to the transmission of malaria, but rainfall and relative humidity were not.	-Only one city was included
				-Cross-correlation		-Socioeconomic factors ware ignored.
				-SARIMA model		
Yang et al. (2010) English [23]	The P.R. China 1981-1995	Yearly growing degree days (YGDD), annual rainfall and relative humidity	Malaria-endemic strata	-A Delphi approach	-Relative humidity was found to be the most important environmental factor, followed by temperature and rainfall. However, temperature was the major contributor of malaria intensity in regions with relative humidity >60%,	-National-level analysis
				-Multiple logistical regression		-Risk maps of malaria based on different climatic factors were developed
				-GIS		
						-Annual indicators were used
Xiao et al. (2010) English [24]	Main island of Hainan province 1995-2008	Monthly average temperature, maximum temperature, minimum temperature, relative humidity and accumulative rainfall	Monthly incidence of malaria	-Cross correlation and autocorrelation analysis	- Temperature during the previous one and two months were observed as major predictors of malaria epidemics.	-Spatial-temporal analysis
				-Poission regression		
				-GIS		-Countermeasure and socioeconomic circumstances ware not taken into account.
					-It was not necessary to consider rainfall and relative humidity to make malaria epidemic predictions in the tropical province.	
Hui et al. (2009) English [25]	Yunnan Province 1995-2005	Monthly average temperature, maximum temperature, minimum temperature, relative humidity and rainfall	Monthly incidence of *P. vivax *malaria Monthly incidence of *P. falciparum* malaria	-Spearman correlation analysis	-Obvious associations between both *P. vivax *and *P. falciparum* malaria and climatic factors with a clear 1-month lagged effect, especially in cluster areas.	-Analysis of both *P. vivax *malaria and *P. falciparum *malaria
				-Temporal distribute analysis		
						-Spatio-temporal analysis
				-Spatial autocorrelation		
					-Minimum temperature was most closely correlated to malaria incidence	
				-Spatial cluster analysis		
				- GIS		
Clements et al. (2009) English [26]	Yunnan Province 1991-2006	Monthly average rainfall, maximum temperature and minimum temperature	Monthly incidence of *P. vivax *malaria Monthly incidence of *P. falciparum *malaria	-Corss-correlation	-Significant positive relationships between malaria incidence and rainfall and maximum temperature for both *P. vivax *and *P.falciparum* malaria	-Analysis of both *P. vivax *malaria and *P. falciparum *malaria
				-Bayesian Poisson regression		
						-Spatial-temporal analysis
				-GIS		
						-Socioeconomic factors were ignored.
					-High-incidence clusters located adjacent the international borders were not explained by climate, but partly due to population migration.	
Tian et al. (2008) English [27]	Mengla County, Yunnan Province 1971-1999	Monthly rainfall, minimum temperature, maximum temperature, relative humidity, and fog day frequency	Monthly incidence of malaria	-ARIMA models	-Temperature and fog day frequency were key predictors of monthly malaria incidence. However, relative humidity and rainfall were not.	-Fog day frequency used *-P. vivax *malaria and *P. falciparum* malaria were pooled together when malaria incidence was calculated.
					-The annual fog frequency was the only weather predictor of the annual incidence of malaria	
Bi et al. (2005) English [28]	Anhui province 1966-1987	Monthly EI-Nino Southern Oscillation Index (ENSO)	Monthly malaria cases	-Spearman correlation	-A positive correlation between ENSO and the incidence of malaria with no lag effect was found.	*-*The impact of ENSO on malaria was analysed -Other meteorological variables were not considered.
						-Only used correlation method
Liu et al. (2006) English [29]	Twenty-one townships of 10 counties in Yunnan province 1984-1993	Monthly minimum temperature, maximum temperature, rainfall, sunshine duration, NDVI.	Monthly incidence of malaria and vector density.	-Principle component analysis	-Remote sensing NDVI and climatic variables had a good correlation with *Anopheles* density and malaria incidence rate.	*-*Both environmental and vector factors were analysed.
				-Factor analysis		
				-Grey correlation analysis		
Bi et al. (2003) English [30]	Sunchen County in Ahui Province 1980-1991	Monthly maximum temperature, minimum temperature, relative humidity and rainfall	Monthly incidence of malaria	-Spearman correlation	-Monthly average minimum temperature and total monthly rainfall, at one-month lag were major determinants in the transmission of malaria.	-Non-climatic factors were neglected
				-Cross-correlation		
						-Only one county considered
				-ARIMA models		
Hu et al. (1998) English [31]	Yunnan Province 1991-1997	Annual rainfall, annual mean temperature	Annual incidence of malaria	- Multiple regression	-Malaria incidence rates are higher in areas with temperature above 18°C, rainfall of more than 1000 mm	-Socioeconomic factors such as income of farmers were taken into account.
				-GIS		
					-Every one degree increase in temperature corresponds to 1.2/10,000 higher malaria incidence and when rainfall increase by 100 mm, malaria will increase to 100.0/10,000	-Annual data were used
Liu et al. (2011) Chinese [32]	Pizhou City, Jiangsu province 2001-2006	Monthly mean temperature, maximum temperature, minimum temperature, rainfall days, relative humidity, evaporation, total cloud cover, sunlight time and low cloud.	Monthly incidence of malaria	-Correlation analysis	-The incidence of malaria was passive relative to temperature, rainfall, relative humidity, evaporation and total cloud cover, but no relation with low cloud and sunlight.	-Various meteorological variables were considered
				-Multiple regression		
						-Only one city was analysed based on a relative short study period
					-The monthly minimum temperature and relative humidity were two major factors influencing malaria transmission.	
Wu et al. (2011) Chinese [33]	Dianjiang county, Chongqing 1957-2010	Monthly mean temperature, maximum temperature, minimum temperature, rainfall days, relative humidity, absolute humidity, duration of sunshine, air pressure and wind speed.	Case counts	-Principal Component Analysis	-Significant associations between malaria incidence and monthly mean temperature, rainfall and duration of sunshine were observed.	-Various meteorological variables were considered
				-Multiple regression		
						-Long-term data from a fifty-four-years period-Only one county considered
					-Temperature was greatest relative to malaria transmission	

Huang et al. (2009) Chinese [34]	Tongbai and Dabie mountain areas, Huibei Province 1990-2007	Monthly mean temperature, maximum temperature, minimum temperature, rainfall.	Case counts	Descriptive study	-Temperature and rainfall were major determinants for malaria transmission and the yearly peak of cases occurred one month after the rainy season.	-Not enough statistical methods
Wang et al. (2009) Chinese [35]	Anhui Province 2004-2006	Annually mean temperature and rainfall NDVI and elevation.	Cases counts	-Principal Component Analysis	-Malaria transmission intensity was positively associated with the NDVI, but negatively associated with minimum temperature, rainfall and elevation.	-Annual indicators were used
				-Logistic regression		-A two-years short period of study.
				-GIS		
Wen et al. (2008) Chinese [36]	Hainan Province May-Oct in 2002	Monthly mean temperature, maximum temperature, minimum temperature, rainfall, relative humidity, land use, land surface temperature (LST) and elevation.	Monthly incidence of malaria	-Spearman correlation	-No associations between meteorological factors and malaria incidence were observed. However, land use, elevation and LST appeared to be good contributors of malaria transmission.	-Various environmental variables were collected
				-Negative binomial regression analysis		
						-A six-month short period of study.
Su et al. (2006) Chinese [37]	Hainan Province 1995	Monthly mean temperature, maximum temperature, minimum temperature, rainfall, relative humidity and NDVI.	Monthly incidence of malaria	-Factor Analysis	-Rainfall and the NDVI may be used to explain the malaria transmission and distribution.	-A one-year short period of study.
				-Principal Component Analysis		
				-Multiple liner regression analysis		
Fan et al. (2005) Chinese [38]	Ailao mountain of Yuxi city in Yunnan Province 1993-2002	Annual man temperature and rainfall	*Anopheles minimus* density	-Correlation analysis	-Significant relationship between malaria incidence and abundance of *Anopheles minimus*. However, no significant correlations between abundance of *Anopheles minimus* and climatic variables.	-No disease data
						-Annual data used
Wen et al. (2005) Chinese [39]	Hainan Province Feb 1995- Jan 1996	NDVI	Monthly incidence of malaria	-Spearman correlation	-Malaria prevalence was highly associated with NDVI value which could be used for malaria surveillance in Hainan province.	-A short study period
				-GIS		
						-No other climatic indicators used
Huang et al. (2004) Chinese [40]	Luodian county 1951–2000 Libo county 1958–2000 Sandu county 1960–2000 Pintang county 1961–2000 Dushan county1951-2000 Guizhou Province	Monthly mean temperature, rainfall, relative humidity	Monthly incidence of malaria	-Correlation analysis	-Significant relationship between malaria incidence and climatic factors, but the influences of different climatic variables were not consistent among the eight study counties.	-Relative long study periods
				-Path analysis		-Direct and indirect effects of climate were analysed by Path analysis
					-The influence of climate on malaria was greater in Libo, Sandu, Dushan counties than in Luodian and Pintang counties	
Gao et al. (2003) Chinese [41]	Yunnan Province 1994-1999	Monthly mean temperature, maximum temperature, minimum temperature, rainfall, relative humidity, rain day, evaporation and sunshine hours	Monthly incidence of malaria	-Back Propagation Network Model	-The efficiency of malaria forecasting was 84. 85% based on meteorological variables.	-Descriptions of associations between malaria and climate was inadequate
						-A five-years short study period
Wen et al. (2003) Chinese [42]	Hainan Province 1995-2000	Monthly average temperature, maximum temperature, minimum temperature, rainfall, relative humidity	Monthly incidence of malaria	-Correlation analysis	-Temperature and rainfall were relative to malaria transmission with various lag times, but relative humidity was not.	-Analysis of high epidemic area and the whole province -Social-economic factors were neglected
				-Stepwise regression analysis		
					-The influence of climatic variables on malaria was more obvious in high epidemic area than that in the whole province	

Huang et al. (2002) Chinese [43]	Jiangsu Province 1973-1983	Monthly rainfall, rain days, relative humidity, evaporation and NDVI	Monthly incidence of malaria	-Correlation analysis	-The NDVI positively correlated with precipitation and relative humidity.	-No temperature data included
				-GIS		-Only correlation method used
					-The NDVI may be a good indicator to predict the distribution and transmission of malaria.	
Huang et al. (2001) Chinese [44]	Gaoan city, Jiangxi Province 1962-1999	Annually average rainfall during April to June, annually average temperature during July to August, annual average rainfall and temperature	Case counts	-Circular distribution method	-Malaria cases increased with increase of average temperature from July to August and rainfall from April to June.	-Annual index were used
				-Descriptive study		
Kan et al. (1999) Chinese [45]	Anhui Province 1969-1999	Annual temperature and rainfall	Annual incidence of malaria	-Descriptive study	-Annual incidences of malaria in 1975, 1977, 1980 in Madian, Lixin County increased with increase of rainfall, while decreased in 1976, 1978, 1981 with decreased rainfall	-Not enough explanation on effects of climate factors on malaria.
						-No statistical methods used
Yu et al. (1995) Chinese [46]	Libo County, Guizhou Province 1958-1993	Monthly average temperature, rainfall, relative humidity	Monthly incidence of malaria	-Correlation analysis	-Positive associations between malaria incidence and climatic factors were observed.	-Relative long study periods
				-Path analysis		
						-Direct and indirect effects of climate were analysed
					-Direct effect of relative humidity was greatest on malaria incidence compared with temperature and rainfall.	

**Table 3:** Characteristics of studies on the association between climatic variables and dengue transmission

**Study & Language**	**Study area & period**	**Data Collection**		**Statistical methods**	**Main findings**	**Comments**
		**Risk factors**	**Disease/vector**			
Wu et al. (2011) English [47]	Liaoning, Hebei, Shanxi, Shaanxi, Sichuan, and Gansu Province 1961-1990	Annual temperature and precipitation, the monthly temperature in January	Distribution data of *Aedes albopictus*	-CLIMEX model	*-Aedes albopictus *have extended their geographic range to areas, which experienced the annual mean temperature below 11°C and the January mean temperature below -5°Cand this may be due to summer expansion	-Risk maps of the potential distribution of *Aedes albopictus *in China were developed -No disease variables included
				-GIS		
Lai et al. (2011) English [48]	Kaohsiung City, Taiwan 2002-2007	Daily air temperature, amount of rainfall, relative humidity, sea surface temperature(SST) and weather patterns of typhoons	Daily number of hospital admissions for dengue fever The incidence of dengue fever, Breteau Index	-Cross-correlation	-Hospital admissions for dengue in 2002 and 2005 were correlated with climatic factors with different time lags, including precipitation, temperature and the minimum relative humidity.	-Both disease and vector factors were considered.
				-Duncan's Multiple Range test		-The impacts of SST and typhoons were discussed.
						-Two case studies of dengue events were included.
				-Spatial auto-correlation analysis		
					-Warm sea surface temperature and weather pattern of typhoons were major contributor to outbreaks of dengue	
				-GIS		
Chen et al. (2010) English [49]	Taipei and Kaohsiung, Taiwan 2001-2008	Weekly minimum, mean, and maximum temperatures, relative humidity and rainfall	Weekly dengue incidence Breteau Index	-Poisson regression analysis	-Weak positive relationships between dengue incidence and temperature variables in Taipei were found, whereas in Kaohsiung, all climatic factors were negatively correlated with dengue incidence	-Both disease and vector factors were considered.
						-Weekly indicators were used
				-Spearman correlation		
					-Climatic factors with 3-month lag, and 1-month lag of percentage BI level >2 were the significant predictors of dengue incidence in Kaohsiung	
Shang et al. (2010) English [50]	Southern Taiwan (Tainan, Kaohsiung and Pingtung) 1998-2007	Daily mean temperature, maximum temperature, minimum temperature, relative humidity, wind speed, sunshine accumulation hours, sunshine rate, sunshine total flux and accumulative rainfall, accumulative rainy hours.	Indigenous dengue cases Imported dengue cases	-Logistic regression	-An increase in imported case favors the occurrence of indigenous dengue when warmer and drier weather conditions are present	-Simultaneously identify the relationship between indigenous and imported dengue cases in the context of meteorological factors
				-Poisson regression		
						-Various climatic data were considered.
Lu et al. (2009) English [51]	Guangzhou City, Guangdong Province 2001-2006	Monthly minimum temperature, maximum temperature, total rainfall, minimum relative humidity,wind velocity	Monthly dengue fever cases and incidences	-Spearman correlation	-Dengue incidence was positively associated with minimum temperature and negatively with wind velocity.	-A relative short 5-years study period.
						-Other environmental and host factors were ignored.
				-Poisson regression		
Hsieh et al. (2009) English [52]	Taiwan 2007	Typhoons, weekly temperature and total precipitation	Weekly dengue incidence Initial reproduction numbers for the multi-wave outbreaks	-Correlation analysis	-A two-wave outbreaks with multiple turning points in 2007 were appeared to be led by the drastic drop in temperature and unusually large rainfall caused by the two consecutive typhoons.	-The important role of climatological events in dengue outbreaks was evaluated.
				-Multi-phase Richards model		
Yang et al. (2009) English [53]	Cixi area, Zhejiang Province (July-October, 2004)	Daily average temperature, rainfall, relative humidity	Case counts	-Descriptive analysis	-No relationship between the incidence of dengue and meteorological factors was observed during the outbreak in 2007	-A short 6-months study period.
						- No statistical methods
Wu et al. (2009) English [54]	Taiwan 1998-2002	Monthly temperature and rainfall Urbanization level	Monthly incidence BI	-Principle components analysis	-Numbers of months with average temperature higher than 18°C and high degree of urbanization were identified as significant indicators for dengue fever infections	-Both climatic variables and socioeconomic factors were considered.
				-Logistic regression		
				-GIS		
Wu et al. (2007) English [55]	Kaohsiung city, Taiwan 1998-2003	Monthly average temperature, maximum temperature, minimum temperature, relative humidity, and amount of rainfall	Monthly incidence Vector density	-Cross-correlation	-Increased incidence of dengue fever was associated with decreased temperature and relative humidity.	-Vector density was analyzed with dengue incidence Only one city was conducted
				-Auto-correlation		
					-Vector density did not found to be a good contributor of disease occurrences.	
				-ARIMA models		
Lu et al. 2010 Chinese [56]	The P.R. China 1970–2000 Guangzhou City and Fujian Province and Ningbo City 2004-2006	Weekly average temperature, maximum temperature, minimum temperature, relative humidity, rainfall and duration of sunshine	Case counts	-Correlation analysis -GIS	-DF outbreaks were significantly correlated with climatic variables with 8–10 weeks lags.	-A risk map of DF outbreaks for China with suitable weather conditions was developed
Yu et al. (2005) Chinese [57]	Hainan Province (before1986, 1986–2001)	Monthly temperature of January Predicted temperature of winter in 2020, 2030 and 2050	Infectious life span of infected mosquito	-Descriptive analysis	-Based on assumptions that temperatures in winter will increase by 1°C and 2°C in 2030 and 2050 respectively, half of or more areas in Hainan Province may be potentially favorable for dengue transmission all the year around by 2030 and 2050.	-Long-term temperature data were collected
				-GIS		-Only considered the temperature
				-Calculation of infectious life span of mosquito in different time periods		-No disease data analysed
Chen et al. (2003) Chinese [58]	Nine cities of Guangdong Province (Dec 2000- Nov 2001)	Monthly mean temperature, relative humidity, rainfall and rainy days	Case counts Breteau index	-Descriptive analysis	-The dengue fever intensity was highly related to increased temperature (>26°C), rainfall and consecutive rainy days (>10 days).	-Study period was short -No statistical methods
Yi et al. (2003) Chinese [59]	Chaozhou City, Guangdong Province 1995-2001	Monthly mean temperature, maximum temperature, minimum temperature, relative humidity, rainfall, rainy days, duration of sunshine	Case counts Breteau index	-Pearson correlation	*-Aedes *density was positively correlated with temperature, rainfall, number of rainy days, duration of sunshine and negatively linked to relative humidity. -Minimum temperature, rainfall and relative humidity are good predictors of *Adeds *density and dengue transmission.	-Various meteorological variables were used -Lag times of climatic factors were not analysed -Both climatic variables and vector factors considered.
				-Stepwise regression		
				-Logistic regression		
Chen et al. (2002) Chinese [60]	Hainan Province 1987-1996	Monthly temperature	Infectious life span of infected mosquito	-Descriptive analysis	-If temperature increase by 1-2°C in winter, Hainan Province will be suitable for dengue transmission all the year around in future due to prolonged infectious life of mosquito.	-Only considered the role of temperature
						-No statistical methods
				-Calculation of infectious life span of mosquito under different temperature		
Zheng et al. (2001) Chinese [61]	Fuzhou City, Fujian Province (2000–2001)	Monthly mean temperature, relative humidity, rainfall	Larva Density, House Index, Container Index, Breteau index, case counts	-Descriptive analysis	-The temperature and rainfall played a considerable role in vector density and dengue transmission, whereas relative humidity showed a little relationship.	-Various mosquito density index used. Study period is relative short

**Table 4:** Characteristics of studies on the association between climatic variables and JE transmission

**Study & Language**	**Study area & period**	**Data Collection**		**Statistical Methods**	**Main findings**	**Comments**
		**Risk factors**	**Disease/vector**			
Lin et al. (2010) English [62]	Linyi city, Shangdong Province 1956-2004	Monthly average temperature, relative humidity, total rainfall. Vaccination	Monthly incidence	-Cross-correlation	-Monthly average temperature and relative humidity with no lag were positively associated with the JE incidence after adjusting for the effect of vaccination.	-Vaccination effect was adjusted, but only treated as a simple binary variable.
				-ARIMA model		
Bi et al. (2007) English [63]	Jinan city, Shangdong Province 1959-1979	Monthly mean maximum temperature, minimum temperature, relative humidity, rainfall and air pressure.	Case counts	-Spearman correlation	-The JE incidence was positively associated with two temperature variables, rainfall and relative humidity, and negatively correlated with air pressure. Lag times were from one to two months	-A potential threshold of the effect of temperature was detected.
				-Poisson regression		
						-The effect of the vaccination was very limited during the study period of this study.
				-The Hockey Stick model		
					-Thresholds of 25.2°C for maximum temperature and 21.0°C were indentified.	
						-Non-climatic factors were neglected
HSU et al. (2008) English [64]	Taiwan 1991-2005	Monthly temperature and precipitation Pig density Vaccination	Case counts	-Poisson regression	-The monthly temperature and precipitation with two months lags and the pig density were significantly associated with JE cases.	-Adjustment for vaccination, pig density and seasonal factors.
					-No significant relationship between vaccination rate and counts of JE cases was found.	
Bi et al. (2003) English [65]	Jieshou County, Anhui Province 1980-1996	Monthly mean maximum temperature, minimum temperature and rainfall	Monthly incidence	-Spearman correlation	-The monthly minimum temperature and precipitation had a significant relationship with JE incidence, with a one-month lag	-Vaccination and other non-climatic factors were neglected
				-Multiple linear regression		
Huo et al. (2011) Chinese [66]	Hebei Province Tianjin City Beijing City Inner Mongolia Shanxi Province 1994-2000	Annual mean temperature, maximum temperature, relative humidity, minimum humidity, rainfall and duration of sunshine	Annual incidence	--Poisson regression	-The annual incidence of JE was found to be positively correlated with annual mean relative humidity and negatively associated with duration of sunshine	-Yearly variables were use
						-Non-climatic factors were neglected
Xu et al. (2009) Chinese [67]	Tongren area, Guizhou Province 1983-2003	Monthly mean temperature, air pressure, relative humidity, rainfall, wind velocity, duration of sunshine	Case counts	-Multiple regression analysis.	-Among various climatic variables, the transmission of JE was only correlated with duration of sunshine.	-Non-climatic factors such as vaccination were not adjusted
						-Only one area were analyzed
Gao et al. (2009) Chinese [68]	Guiyang City, Guizhou Province 1956-2005	Annual mean temperature and precipitation Monthly mean temperature and precipitation of June, July and August.	Annual incidence	-Descriptive analysis	-Temperature and precipitation were correlated with the incidence of JE, especially in July.	-Fifty years long-term data were collected
						-Non-climatic factors such as vaccination were ignored.
Liu et al. (2008) Chinese [69]	Kaijiang County, Sichuan Province 1975-1993	Mean temperature, relative humidity, rainfall, duration of sunshine during November and December, July and August, January and June respectively.	Annual incidence	-Correlation analysis	-Duration of sunshine and temperature were most closely associated with JE incidence.	-Only one county was analyzed -Annual indicators were used
				-Grey correlation analysis		
						-Non-climatic factors such as vaccination were ignored.
Qu et al. (2006) Chinese [70]	Chaoyang City, Liaoning Province 1981-1994	Annual mean air pressure, precipitation, air temperature, ground temperature, maximum air temperature, minimum ground temperature, evaporation and extreme maximum and minimum temperature	Annual incidence	-Correlation analysis	-The JE incidence was negatively correlated with air pressure, and positively correlated with evaporation, maximum temperature and extreme maximum temperature.	-Various meteorological factors were applied
				-Back propagation artificial neural network		-The predictive ability of the BP neural network model is not very strong.
Zhang et al. (2004) Chinese [71]	Dali, Yunnan Province 1992-2001	Mean temperature in May, rainfall in September, annual mean temperature, rainfall estimated vaccination coverage, paddy field areas	Annual incidence	-Correlation analysis	-The annual JE incidence was found to be correlated with temperature and rainfall. No relationships between the JE incidence and estimated vaccination, as well as paddy field areas were found.	-Use of approximate estimated vaccination data
				-Multiple regression		-Data of paddy field areas were collected.
Liu et al. (2003) Chinese [72]	Chaoyang City, Liaoning Province 1983-2002	Mean temperature and rainfall during June and August, annual mean rainfall	Annual incidence	-Correlation analysis	-The annual JE incidence was just correlated to the rainfall in July among climatic factors selected.	-Non-climatic factors such as vaccination were ignored.
				-Multiple regression		
						-Annual incidence was used
Shen et al. (2002) Chinese [73]	Shanghai 1952-1997	Monthly temperature of June, July and August respectively, total rainfall of June and July Areas of rice field, pig rising, mosquito density, vaccination rate	Annual incidence	-Descriptive analysis	-No obvious relationships between JE incidence and climatic factors and areas of rice field as well as pig rising were observed, implying that the decrease of JE incidence during study period may be due to massive vaccination conducted in Shanghai.	-Both climatic and non-climatic data were collected
						-Climatic variables only in three months were analysis
Zhang et al. (1997) Chinese [74]	Henan Province Not specific	Temperature, rainfall Elevation	Case count JE incidence	-Correlation analysis	-The JE incidence was positively correlated with temperature and rainfall, but decreased with increased elevation.	-The impact of vaccination was ignored
						-Data collection was not described clearly
Feng et al. (1996) Chinese [75]	Fengyi of Eyuan County, Dali, Yunnan Province 1991	Monthly mean temperature and rainfall	Monthly incidence	-Descriptive analysis	-The monthly incidence was found to be related to monthly temperature and rainfall	-Only one year data was analysed

Study sites mainly included Yunnan Province (n = 9), Hainan Province (n = 8), Anhui Province (n = 7), Taiwan (n = 7), Guizhou Province (n = 5), Shandong Province (n = 3), Guangdong Province (n = 3), Henan Province (n = 2), Hubei Province (n = 2), Jiangsu Province (n = 2), and Liaoning Province (n = 2). Other locations included Jiangxi Province, Hebei Province, Shanxi Province, Shaanxi Province, Sichuan Province, Gansu Province, Zhejiang Province, Fujian Province, Beijing Municipality, Chongqing Municipality, Tianjin Municipality, Inner Mongolian Autonomous Region, and Tibetan Autonomous Region. However, there was no study conducted in some regions where mosquito-borne diseases are endemic, such as Guangxi and Hunan Province.

All included studies examined the relationship between climatic variables and mosquito-borne diseases. Of these, only 6 studies evaluated of the impacts of both meteorological factors and other relevant determinants, such as urbanization, agriculture and vaccination [51,54,64,71,73]. Several studies used ecological proxy indicators including Normalized Difference Vegetation Index (NDVI) and South Oscillation Index (SOI) as risk variables [28,29,35,37,39,43]. Two studies explored the influence of typhoons on outbreaks of dengue fever [48,52]. The main outcome indicators evaluated in this review were case count and incidence rate. Several studies collected data on entomologic factors [38,47,48,60]. Only 7 studies evaluated both disease and mosquito data together [21,48,49,54,55,59,61].

A variety of methods were used to determine the effects of climatic variables on diseases and mosquitoes. Among them, 9 employed spatial study designs [23,31,35,39,43,47,48,54],[56], 6 time-series analyses [20,22,27,30,55,62], and 4 spatial-temporal methods [19,24-26]. Simple comparisons between climate, disease and/or vector data were applied in 10 descriptive studies [34,45,53,57,58,60,61,68],[73,75]. Correlations and multiple regressions (e.g logistic and Poisson regression) were widely conducted among selected articles to examine associations between weather parameters and mosquito-borne diseases. Time-series models including autoregressive integrated moving average (ARIMA) model and seasonal ARIMA model were mainly used in recently published studies. Risk maps of mosquito-borne diseases and vector distribution in different regions were also present in some articles using spatial analysis with Geographic Information System (GIS) [23,47,56]. Other statistical methods used included study designs utilizing Principle Component Analysis (PCA), Back propagation artificial neural network and CLIMEX model etc.

### Associations between climatic variables and mosquito-borne diseases

#### Malaria

Despite considerable reductions in the overall burden of malaria in the 20th century, this ancient disease still represents a significant public health problem in China, especially in the southern and central regions. In 2010, 7,855 diagnosed malaria cases and 34,082 suspected cases with 19 deaths were reported in 1191 counties of 239 Provinces/Municipalities/Autonomous Regions in China. The annual incidence was 0.66/10,000 population [76]. Only sixteen percent of malaria cases were caused by *Plasmodium falciparum*[76] mostly occurring in Yunnan Province, which is located in southern China. Yunnan remains a hypo-endemic region with persistent cases of both *P. falciparum* and *P. vivax* malaria.

To identify risk factors related to climate change and its role in malaria transmission, a series of studies were conducted in mainland China to investigate the relationships between meteorological variables and malaria [19-46]. Except for a single study that found no association, all studies showed correlations between climatic variables and malaria in different locations and study periods in China. The contradictory study likely resulted from a short (6-month) study period [36]. In Yunnan province, two studies were conducted in 2009 to clarify potential risk factors of malaria transmission [26,31]. Clements et al. (2009) demonstrated that for *P. vivax* the relative risk appeared to cycle every 3 to 4 years, whereas for *P. falciparum* the pattern was less regular [26]. Hui et al. (2009) found that the influence of meteorological variables on *P. vivax* malaria was greater than that of *P. falciparum* malaria, especially in cluster areas, indicating *P. vivax* malaria may be more climate-sensitive [31].

Almost all of these analyses identified a positive association between temperature indices and malaria transmission. Some studies also reported that temperature was the most important climatic determinant in the transmission of malaria. For example, a study conducted in Jinan, which is a temperate city in northern China, showed that a 1°C rise in maximum temperature may be related to a 7.7% to 12.7% increase in the number of malaria cases, while a 1°C rise in minimum temperature may result in approximately 11.8% to 12.7% increase in cases [22]. Zhou et al. (2010) revealed that temperature was a key meteorological factor correlated to malaria incidence, implying the potential role of global warming in malaria re-emergence in central China early in the 21th century, especially in Anhui, Henan and Hubei Provinces along the Huang-Huai River [21]. However, the association between temperature variables and malaria incidence may not be constant year-round. Tian et al. (2008) emphasized the stronger effect of minimum temperature on malaria incidence in the cool months in the rain forest area of Mengla County, Yunnan province, indicating increased risk of transmission as a result of warmer winters [27]. Although global warning could make more areas climatologically suitable for malarial transmission; because higher temperature promotes mosquito development, virus replication and feeding frequency of mosquitoes, extreme high temperature can also restrict the growth of mosquitoes and reduce the spread of malaria. Typically, temperatures lower than 16°C or higher than 30°C have a negative impact on the development and activities of mosquitoes [30].

High relative humidity is expected to prolong the life of the mosquito enabling it to transmit the infection to several persons. Correlations between relative humidity and malarial transmission were also detected in some regions in China [19,20,23-25,30,32,39,40,46]. According to results from regression models based on 15 years of data, Yang et al. (2010) found that relative humidity was more important than rainfall and temperature in addressing the climate-malaria relationship in China [23]. In Motou County of Tibet, Huang et al. (2011) found that relative humidity, which was greatest relative to malaria incidence among meteorological variables as it is a result of temperature, rainfall and other climatic indicators and influenced the activity of mosquito directly such as biting rate and breeding rate [20]. The distribution of mosquitoes, which also is also dependent on relative humidity, determines the extent of malarial spread. Thus, no malaria transmission occurs where the monthly average relative humidity is lower than 60% [23]. Conversely, it was reported that relative humidity is not a restricting factor in areas where it is higher than 60%, but temperature then becomes the major driver [23]. For example, no association of relative humidity and malaria transmission was detected in Hainan province [24,42] and the tropical rain forest regions of Yunnan provinces [27], where the relative humidity throughout the years is much higher than 60%. This indicates that it is not necessary to consider humidity when making malaria epidemic predictions in areas of consistently high humidity.

The impact of precipitation on malaria transmission is inconsistent across geographical locations in China. Some studies indicated that rainfall was closely correlated to malaria incidence [19-21,23-26,30-35,37,39,40], whereas some failed to detect such an association [22,27,36]. Rainfall not only provides the medium for the mosquito life cycle, but is also related to high humidity, thereby enhancing mosquito survival. Using Bayesian hierarchical models, Huang et al. (2011) showed that the way rainfall influenced malaria incidence in central China was different from other climatic factors. This implies that malaria incidence is more sensitive to rainfall compared to other meteorlogic variables [20]. However, the association between mosquito abundance and rainfall is non-linear. Excessive rainfall often leads to small puddles serving as mosquito breeding sites and therefore increases malaria transmission. But heavy rain may destroy existing breeding places and flush the eggs or larvae out leading to reduced transmission [25,30]. A negative effect of rainfall on malaria spread was detected by Wang et al. in Anhui province, which identified that every 1 mm annual rainfall increase corresponds to 27% decrease of malaria cases [35]. Fog precipitation, as another important source of water in many mountainous and coastal regions, was first found to be a predictor of malaria in the tropical rain forest area of Mengla County, southwest China by Tian et al. [27].

In some studies ecological proxy indicators such as Normalized Difference Vegetation Index (NDVI) [29,35,37,39,43] and South Oscillation Index (SOI) [28] were also analyzed for the detection of the climate-malaria relationship. For example, in southeastern Yunnan Province, remote sensing NDVI was found to be a sensitive evaluation index of Anopheles density and malaria incidence rate by using grey correlation analysis [29]. The EI Nino-Southern Oscillation (ENSO) represents a periodic variation in the atmospheric conditions and ocean surface temperatures of the tropical Pacific and was determined to have a positive influence on malaria incidence in Anhui province, China [28].

The effect of climatic factors on mosquito-borne diseases including malaria is not immediate and usually results in a lag-time due to the life cycle of the vector and the parasite [20]. Lag times of different climate indicators were analyzed in some studies on different geographical and temporal scales. For example, Zhou et al. (2010) reported a 75.3% change in monthly malaria incidence was correlated with the average monthly temperature, the average temperature of last two months and the average rainfall of current month in central China [21]. In Yunnan Province, obvious associations between both *P. vivax* and *P. falciparum* malaria and climatic factors with a clear one-month lagged effect were found [25]. It is essential to take lag effect into account in addressing the impact of climate change at a local level because it provides important information for early detection and warning for mosquito-borne diseases.

#### Dengue fever

Dengue fever is the most common arboviral disease in the tropics and subtropics, and about 2.5 billion people live in regions at risk for dengue transmission [77]. Since the first recorded outbreak of dengue fever in Foshang City in 1978, dengue occurs frequently in southern China, including Guangdong, Guangxi, Hainan, Taiwan, Fujian, Zhejiang and Yunnan [78]. In China, *Aedes albopictus* is the most important mosquito in dengue transmission in China. Due to its wider geographic distribution it could be responsible for recent dengue outbreaks in Guangzhou and Zhejiang Province [79]. As another major vector of dengue virus, distribution of *Aedes aegypti* which was previously considered only in the coastal areas of the tropical zone below 22° N latitude has already extended into regions of 25° N latitude, such as Yunnan Province [80].

Recently, the impacts of climate change on dengue transmission and dengue vector distribution in China have been evaluated and identified in limited studies [47-61]. Although meteorology alone does not initiate dengue epidemics and it is reported that there appears to be a smaller climatic effect on this disease than occurs with other arboviruses [81]. Temperature, rainfall and relative humidity were considered major meteorological determinants in most of studies, whereas one study from Cixi, Zhejian Province reported no correlation between dengue outbreaks and climatic factors [53]. This is perhaps due to the non-endemic nature of dengue in Cixi and a short (4-month) study period.

Although results of studies with varied temporal, spatial or spatiotemporal approaches are not consistent in terms of the effect of temperature [82,83], historical data do suggest that temperature plays an important role in vector competence and dengue transmission [84-86]. Some studies in China have also identified an obvious association between temperature variables and vector distribution, dengue outbreaks and distribution. In Hainan Province, Chen et al. (2002) revealed that under global warming conditions, Hainan would be suitable for continuous dengue transmission with dengue fever cases year-round [60]. Similarly, Yu et al. (2005) reported that over half of the area of Hainan would be favorable for year-round dengue transmission by 2030 and 2050 based on predicted winter temperatures [57]. Using the CLIMEX model, Wu et al. found that due to summer expansion, *Ae. albopictus* have extended their geographic range to areas which experienced an annual mean temperature below 11°C and a January mean temperature below -5°C. This finding highlights that most provinces and cities in China now support survival and development of *Ae. albopictus* and risk the occurrence of dengue fever or the establishment of dengue virus in the mosquito population [47]. In subtropical Taiwan, it was found that every 1°C increase of monthly average temperature could lead to 1.96 times increase of the total population at risk for dengue fever transmission, indicating that a slight increase in temperature could result in epidemics of this disease [55]. Therefore, climate change, particularly a warming trend, increases the land area suitable for disease vectors,altering or increasing dengue fever distribution. Furthermore, other climatic indicators such as rainfall, relative humidity and wind velocity together with temperature can be significant predictors of dengue incidence. For example, Lu et al. (2009) showed that in Guangzhou City minimum temperature and minimum humidity, at a lag of one month, were positively associated with dengue incidence, whereas an obvious negative effect of wind velocity on dengue cases was observed in the same month [51].

Unfortunately, less work was conducted to relate dengue outbreaks and climatological events in mainland China. Several studies in Taiwan reported that typhoons remain an important factor affecting vector population and dengue fever [48,52]. Lai (2011) found that two outbreaks of dengue and increasing vector population in Kaohsiung, Taiwan in 2002 and 2005 were exacerbated by hot and wet climate conditions caused by warm sea surface temperatures and typhoon weather patterns [48]. Typhoons could result in massive rainfall, high humidity and water pooling resulting in mosquito breeding sites [48]. Conversely, a sharp drop in temperature and substantial rainfall caused by frequent typhoons may contribute to a temporary reduction in dengue infections [52]. More research on the impact of extreme climatic conditions such as floods, droughts, typhoons and storms on mosquito-borne diseases are needed in China.

Along with climatic drivers, many site-specific variations in some factors affecting dengue transmission, such as mosquito density, imported cases and other environmental factors were also identified and highlighted in a few studies in China [48-50,53,55,59]. Indices such as the Breteau Index (BI), Housing Index (HI) and Container Index (CI) were traditionally employed to determine mosquito density. In Kaohsiung city, Taiwan, the BI, which indicates the number of positive containers per 100 houses, in addition to local climatic factors were found to be good predictors of dengue incidence [49]. Similarly, Lai et al. (2011) demonstrated that the number of dengue fever admissions in Kaohsiung city was significantly correlated with BI with a time lag of 32 and 22 days during both summer and autumn in 2002 and 2005, respectively [48]. From a spatial standpoint, one study examined the relationships among weather profiles, environmental factors of interest, socioeconomic factors and geographical distributions of dengue fever and showed the number of months with average temperature higher than 18°C and the level of urbanization were significantly associated with dengue fever risk at the township level in Taiwan [54]. Using logistic and Poisson regression models, Shang et al. (2010) emphasized the importance of imported case and favorable climate conditions in the initiation of dengue epidemics, also highlighting that the development of an early warning surveillance system, utilizing relevant meteorological information, will be an invaluable tool for prevention and control of dengue fever [50]. Other environmental and host factors, such as intervention measures and human risk behaviors, also influence mosquito populations and the extent of dengue spread. Thus, more work should be conducted in the future for a better understanding of these complex interactions.

#### Japanese Encephalitis

Japanese encephalitis (JE), a mosquito-borne viral disease, is mostly transmitted by *Culex tritaeniorhynchus* in China, with pigs as a reservoir host and source of infection [87]. Due to mass vaccination the 1980s and improved economic circumstances in China, the morbidity and mortality due to JE has declined gradually each year [88]. Over a 6-year period between 2000 and 2005, the annual incidence decreased from 0.9489/100,000 to 0.3898/100,000 [88]. Similar findings were also observed in Taiwan. Since mass vaccination was implemented in 1968, the incidence of JE has declined from 2.05/10000 to 0.03/10000 in ten years from 1967 to1997 in Taiwan [89]. However, JE is still one of an acute epidemic disease posing a threat to public health, and it has recently spread to new territories [88]. In 2009, JE virus was isolated from *Cu. tritaeniorhynchus* mosquitoes collected in Tibet, indicating that JE virus has extended its geographical range to a region that was previously non-endemic due to high elevation [90]. Such trends in geographical spread of JE were also recently reported in other countries such as Australia [91-93]. Highly endemic areas of JE in China include Sichuan Province, Guizhou Province, Chongqing Municipality, Yunnan Province and Shaanxi Province, which are mainly located in southwest and central China. The five provinces account for 50% of the total cases nationwide [88].

Global warming might change temperature and rainfall patterns [94,95], which may affect the development and infection capacity of both the mosquito and the virus. Relative humidity is also important in the transmission of JE because mosquitoes can survive longer and disperse further in areas with suitable relative humidity [96]. Studies in different areas of Asia have also shown the likely influence of climate on the incidence of JE [97-99]. However, little research has been conducted to examine the effect of climatic variables, along with mass vaccination and other non-climatic drivers in China. Bi et al. (2007) have identified positive relationships between climatic variables (monthly maximum temperature, minimum temperature and total rainfall) and JE transmission in a rural region of Anhui Province [65] and a metropolitan area of Shangdong Province [63] where no rice was grown and the role of pigs in disease transmission was not fully understood [63]. Unfortunately, the effects of vaccination on JE control in the two areas were very limited during the study periods. In the metropolitan area of Jinan city, a potential threshold of the effect of temperature on JE was also detected by the Hockey Stick model which is based on the assumption that temperature has no effect on JE cases until a threshold value. When the monthly mean maximum temperature was higher 25.2°C or the minimum temperature was over 21.0°C, an obvious increase in JE cases occurred [63]. These findings are consistent with the threshold temperature detection in previous Chinese studies [87]. Using ARIMA models, Lin et al. (2011) suggested that monthly average temperature and relative humidity at 0-month lag were positively associated with JE incidence in Linyi, another city of Shangdong Province after adjusting for mass vaccination in this area [62]. Time lag-0 of climate variables was perhaps because the behaviour of pig breeding in Linyi, along with the high density of mosquitoes help to shorten the transmission cycle [62]. With adjustment of more interactional factors including seasonal pattern, time trend, pig density, 23 geographic areas representing location of farm and paddy cultivation, and vaccination coverage, HSU et al. (2008) identified the significant effects of monthly temperature and rainfall with two months lag on the monthly occurrence of JE in Taiwan [64]. Similarly, temperature and rainfall were two significant determinants of JE spread with control of vaccination coverage and paddy field areas [71].

Few analyses, without controlling for non-climatic factors that potentially affect JE transmission, also reported associations between different climatic variables such as temperature, rainfall and JE annual incidence by correlation and regression analyses [66-69,72,74]. Using multiple stepwise regression, Xu et al. (2009) found that among various meteorological indicators the transmission of JE was only correlated with duration of sunshine in Tongren area, Guizhou Province [67]. Similarly, a close relationship between sunshine and annual incidence of JE was also reported by Huo et al. [66] and Liu et al. [69] in north China and Kaijiang county of Sichuan Province in Southwestern China, respectively. Applying correlation analysis and back propagation artificial neural work, the annual JE incidence was found to be negatively correlated with mean air pressure, and positively correlated with mean evaporation, maximum temperature and extreme maximum temperature [70]. In future research investigating the influence of climate change on JE transmission, important factors should be measured and controlled for, such as social- economic status, population immunity (including vaccination), mosquito control measures, pig rising, areas of rice field and the virulence of the virus. Moreover, research is also needed in sporadic- and meso- endemic areas, such as Jiangxi Province, Hunan Province, Fujian Province and Guangdong Province.

### Implications for adaptation in China

The potential effects of climate change on the spatial and temporal distribution of mosquito-borne diseases and vectors in China have been summarized in this review. Furthermore, the synthesis of the literature shows an urgent need for improving current control policies and developing targeted adaptive strategies in China to address mosquito-borne disease. Although some health benefits have also been achieved through mitigation policies in China, adaptation, preparing to manage some of the unavoidable effects of climate change on human health, is another important response strategy [100]. Based in part on recommendations and established approaches in recent studies of adaptation to climate change [81,101-105], as well as on the status of mosquito-borne diseases in China, we highlight five principles to guide timely development of adaptation mechanism to reduce the adverse impacts of climate change on the control of mosquito-borne diseases. These guidelines may also be applicable in addressing the threat to other health outcomes from climate change.

#### Improving current surveillance and monitoring systems integrated with climate-sensitive conditions

In recent years, the Chinese government has paid great attention to the prevention and control of mosquito-borne diseases. Since 2004, cases of infectious diseases have been electronically recorded and the data collected at the national level by the Chinese Center for Diseases Control and Prevention (China CDC). This important step means the disease surveillance system is more sensitive and efficient than in previous years. However, unavailability of good quality long-term data sets has hampered our understanding of the likely impacts of climate change on mosquito-borne diseases and vector distribution. Kovats et al. (2001) have pointed out that climate change and health researches require at least 30 years of data because short- and medium- term associations may not provide an accurate picture of the impact of climate change occurring over many decades [106]. In China, imperfect data collected by passive surveillance systems restrict such climate-health relationship analysis. For example, onset dates rather than notification dates are not available, which may lead to considerable information bias. According to a national report in 2005, it was also estimated that only 1/18 (5.6%) malaria cases in China are reported [107]. Furthermore, lack of routine data collection of vectorial indicators has restricted our understanding of real geographic and temporal distributions of mosquito vectors.

Under the circumstance of the changing climate, truly effective surveillance systems and monitoring systems can be used to identify changes in the range and incidence of diseases; determine whether these changes are to be the result of climate change; assist the development of response measures and develop hypotheses about the climate-health relationship [108]. We believe that there are also many gaps for improving current surveillance and monitoring of infectious diseases in China in response to climate variation. Surveillance for early detection of epidemics of mosquito-borne diseases based on readily climatic data, such as daily temperature records, in combination with other interactional factors are of paramount importance [108]. For example, an effective early warning system for outbreaks of mosquito-borne diseases based on predicted extreme weather conditions, such as extreme temperature or rainfall, can be considered as an immediate opportunity for adaptation by strengthening the preparedness of emergency response before periods of high-risk [100]. Careful tracking of imported cases, in conjunction with relevant meteorological data, is also of assistance in providing earlier warning signals for emerging indigenous epidemics [109,110]. In short, coherent surveillance systems integrated with climate-sensitive conditions are urgently needed to improve scientific knowledge about the health risks of climate change, and to prioritize needs for intervention and adaptation options.

#### Focusing adaptation strategies and policies on vulnerable communities

Some populations and geographical regions will be particularly vulnerable to climate change. Although climate change is a global threat to public health, it is well acknowledged that poorer nations and communities who have contributed least to greenhouse gas emissions are most vulnerable to the effects [111]. Higher malaria risk in China has also been associated with poverty, poor quality housing, unhygienic surroundings and agricultural activities in rural and remote areas. Chinese farmers in rural regions who usually work in fields and sleep in the open are at higher risk of mosquito biting especially in summer and autumn, when the peak times of malaria occurred due to favorable climate conditions and active propagation of mosquitoes. However, urban populations may also share some increased vulnerabilities in the context of climate change. For example, populations living in cities located on the coast or on small islands may be particularly vulnerable to frequent rainfall and storms and are also exposed to changes in the spread of mosquito-borne diseases, such as Taiwan. Additionally, metropolitan regions may be experiencing two types of warming trends;warmer temperatures caused by the urban heat island effect as well as global climate change, which could make more urban areas suitable for the transmission of mosquito-borne diseases by reducing development times, increasing survival probabilities and biting frequency for the mosquitoes [102,112].

Due to limited funding, resources, and time, effective adaptive action is required to protect the most vulnerable individuals and communities, due to geographic locations and low adaptive capacity, from inevitable effects of climate change on mosquito-borne diseases. Firstly, better identification of real vulnerable groups needs to be based on more comprehensive factors, such as political rigidity, population growth, poverty, culture, dependency, geographic isolation, population immunity and human perceptions, behaviours and activities etc. This requires both qualitative and quantitative assessing methods in future adaptation research. The next step is the development and implementation of timely and efficient adaptive strategies in those targeted communities by collective coordination of all relevant sectors. Adaptive options may include expanding mosquito control, improving vaccination coverage, enhancing existing elimination programs and conducting health education programs in a relative short term, and the establishing an early warning system, improving housing quality, strengthening preparedness and response of extreme weather events (e.g. better and adequate urban drainage systems) in a long run.

#### Strengthening the capacity of public health system to adapt climate change

In the Chinese context, an efficient network system of mosquito-borne diseases control has been established and CDCs at national, province, and city level take the major responsibility in the network. Although the public health system has credible skills and experience ranging in disease control and prevention, public health management and emergency preparedness services, the status of the resources and capacity of mosquito-borne diseases control at local levels is not optimistic, especially in rural counties, townships and communities. For example, Chen et al. (2010) pointed out that lack of additional funding, additional staff, staff straining and equipment currently has become the major hamper of improving ability of local public health sectors in malaria surveillance, diagnosis and treatment, and mosquito control [113].

The climate change will have local impacts, and a significant share of dealing with the adverse impacts of the change will fall on local public health arena. Making climate change adaptation a priority for local public health sectors, however, is challenged in current China due to a chronic lack of resources and limited awareness and knowledge about health impacts of climate change. Disappointedly, health implications of climate change have largely neglected in not only developing countries but developed ones, compared with its energy, economic and environmental implications. For example, shortage of public health professionals and small part of research funding of climate change for public health were reported among public health department directors in the U.S., which are also a major challenge for China in adapting the changing climate [114]. Moreover, inaccessible information and training on health influences of climate change may restrict awareness, knowledge, attitude and decisions of local public health departments. In brief, adaptation to climate change will require the public health system has a key leadership role to take in health adaptation strategy making and implementation in China. Stable funding, additional staff and better information access will be needed to best prepare the public health sectors to manage the health risks associated with climate change.

#### Developing multidisciplinary approaches sustained by a new mechanism of intersectoral coordination and collaboration

What makes addressing the range and magnitude of health impacts of climate change even more difficult is unavoidable complexities and uncertainties in multi-factorial causal webs. We can not deal with climatic variables or health outcomes or any other potential interacting drivers in isolation and need to integrate scientific knowledge from various disciplines to tackle these interactions by developing a better collaborated mechanism across all relevant governmental and non-governmental sectors and institutions which are responsible for the prevention and control of climate-sensitive disease [101,112]. Although the existing national plans and policies about climate change clearly indicate that adaptation to health burdens from climate-sensitive diseases is a multiple-sector responsibility, mechanisms of intersectoral decision making and coordination do not yet operated to guarantee free and open exchange of information, adequate compliance and participation, ongoing financial and technological support.

For the control and prevention mosquito-borne diseases under condition of climate change, it is even more urgent to produce multidisciplinary insights from diverse public and private sectors. Although some health risks can be reduced largely by health sector interventions such as surveillance, mosquito control, spraying, vaccination, sanitation activities and health education, many adverse impacts require concerted adaptive options with other relevant sectors such as meteorology, environmental, urban designing and planning, water, agriculture and housing [101]. Unfortunately, health sectors in China which invest greater financial and resources support, are often the only ones responsible for disease prevention and control. Therefore, there is a particular need for a multidisciplinary approach sustained by ongoing intersectoral coordination and collaboration, which not only allow us to have a bettering understanding of the complex climate-health relationship, but will provide integrated and practical adaptive strategies to minimize climate-sensitive disease impairments, and further influence policy-formulation and decision-making [115,116].

#### Promoting awareness and mobilization of the public and individuals

An important step in the development and promotion of successful local adaptation options is raising public and professional awareness. Governments, institutions, and organizations play indispensable roles in those adaptive actions but so do the public and individuals if they are receptive to behavior change to adapt to a world altered by climate change [101]. It is well acknowledged that the perceived risk of climate change in the population is the strongest motivator of health behaviour change, that is, it is only when individuals feel vulnerable and threatened to the impacts of climate change that they will take autonomous adaptation seriously [117]. In China, climate change has traditionally been treated as an environmental threat rather than a public health issue. We highlight that awareness programs about the health aspects of climate change are urgently needed, coupled with high-quality baseline investigations to examine public perception of adverse health effects from climate change in China.

Mobilization of the public to adapt to climate change also depends on availability of information about effective adaptation measures as well as social capacity to deal with these problems. A recent cross-sectional survey conducted in the U.S. showed that people who report knowledge of the necessary information to prepare for adverse health impacts of climate change were more likely to have an adaptive plan for their household [118]. For the prevention of mosquito-borne diseases, scientific knowledge and information, such as risk behaviour and self-protection measures, should be rapidly dispersed during high-risk periods to strengthen the adaptive capacity of the public. For example, relevant health intervention campaigns can be conducted to warn and educate local communities to change personal behaviour, e.g. use of mosquito nets in the field at peak time of mosquito-borne diseases; cleaning living conditions as soon as an increase in cases; emptying artificial containers with stagnant water timely. Moreover, better implementation of planned adaptation to climate change requires good social capability which depends on resource availability and cultural acceptability, indicating that successful adaptation strategies much be suitable for local conditions and accepted by local populations [101].

## Conclusions

This study included scientific evidence of impacts of climate change on the transmission of mosquito-borne diseases, resulting in increase in incidence and geographic spread in China. Variability in temperature, precipitation, wind and extreme weather events has been observed to be linked with changes of spatial and temporal distribution of malaria, dengue fever, Japanese encephalitis in some regions in China. However, research to date has limitations and challenges in attributing changes in the status of mosquito-borne diseases to climate change. Potential adverse effects heighten the urgent need to conduct more high-quality research for assessing risks of these climate-sensitive vector-borne diseases, to improve current control policies from a weather-based direction, and to develop targeted policies for adapting short-term and long-term climate shifts in China.

Based on summarization of what is known about the likely impacts of climate change on these diseases in China we highlight five principles to guide policy formulation to enhance adaptation mechanism to reduce the adverse impacts of climate change on the control of mosquito-borne diseases. These recommendations include: 1) improving current surveillance and monitoring systems integrated with climate-sensitive conditions; 2) focusing adaptation strategies and policies on vulnerable communities; 3) strengthening public health system capacity to adapt to climate change; 4) developing multidisciplinary approaches sustained by a new mechanism of intersectoral collaboration; and 5) promoting awareness and mobilization of the public as well as health and other professionals.

## Abbreviations

IPCC: Intergovernmental Panel on Climate Change; CHKD: China Hospital Knowledge Database; NDVI: Normalized Difference Vegetation Index; SOI: South Oscillation Index; ARIMA: Autoregressive integrated moving average model; GIS: Geographic Information System; PCA: Principle Component Analysis; P. vivax: *Plasmodium vivax*; P. falciparum: *Plasmodium falciparum*; ENSO: EI Nino-Southern Oscillation; BI: Breteau Index; HI: Housing Index; CI: Container Index; JE: Japanese encephalitis; CDC: Center for Diseases Control and Prevention.

## Competing interests

The authors declare that they have no competing interests.

## Authors’ contributions

LB and QL designed the study and carried out the literature search. LB and LCM reviewed included studies and wrote the paper. All authors read and approved the final manuscript.
